# Precision medicine becomes reality—tumor type-agnostic therapy

**DOI:** 10.1186/s40880-018-0274-3

**Published:** 2018-03-31

**Authors:** Li Yan, Wei Zhang

**Affiliations:** 1 US Chinese Anti-Cancer Association, Los Angeles, Martinez, CA 94553 USA; 2 Beijing Cancer Hospital and Institute, Peking University School of Oncology, Beijing, 100142 P. R. China; 3 Cancer Genomics and Precision Oncology, Wake Forest Baptist Comprehensive Cancer Center, Winston-Salem, NC 27157-1082 USA

**Keywords:** Precision medicine, Anti-programmed cell death-1, Microsatellite instability-high, Deficient DNA mismatch repair

## Abstract

Precision medicine just witnessed two breakthroughs in oncology in 2017. Pembrolizumab (Keytruda), Merck’s anti-programmed cell death-1 (PD-1) monoclonal antibody (mAb), received accelerated approval in May 2017 by the US Food and Drug Administration for the treatment of adult and pediatric patients with unresectable or metastatic solid tumors that have been identified as having microsatellite instability-high (MSI-H) or deficient DNA mismatch repair (dMMR). Shortly after, nivolumab (Opdivo), Bristol-Myers Squibb’s anti-PD-1 mAb, gained an accelerated approval in August 2017 for adult and pediatric patients with MSI-H or dMMR metastatic colorectal cancer that has progressed after standard chemotherapy. These regulatory approvals marked an important milestone that a cancer treatment may be approved based on a common biomarker rather than the anatomic location in the body where the tumor originated, and therefore established a precedent for tumor type-agnostic therapy. In the 2017 American Society for Clinical Oncology annual meeting, larotrectinib (LOXO-101), Loxooncology’s oral, potent, and selective inhibitor of tropomyosin receptor kinases (TRK), demonstrated unprecedented efficacy on unresectable or metastatic solid tumors with neurotrophic tropomyosin receptor kinase (NTRK)-fusion proteins in adult and pediatric patients. Both the anti-PD-1 mAbs and the TRK-targeting therapies share some basic features: (a) biomarker-based, well-defined rare patient population; (b) exceptionally high clinical efficacy, e.g., near 40% overall response rate (ORR) for pembrolizumab across 15 tumor types with MSI-H/dMMR and 75% ORR for larotrectinib across more than 12 tumor types with NTRK-fusion proteins; (c) durable responses lasting at least 6 months with complete responses observed; and (d) parallel development in adult and pediatric populations. With increasing accessibility to genetic analysis tools such as next-generation sequencing, tumor type-agnostic therapy has become a reality, both during clinical development and in clinical practice. Adjustments in our approaches to developing new anti-cancer drugs and to adopting these new cancer treatments in clinical practice need to occur in order to prepare ourselves for the new era of precision medicine.

## Background

Traditional anti-cancer drug development follows a well-established paradigm, e.g., taking a new molecular entity (NME) into individual tumor types that are hypothesized to be dependent on the biological mechanism targeted by the NME. The introduction of biomarkers into drug development has helped focus clinical testing of these NMEs on subpopulations that may be more sensitive to the pharmacodynamic modulations by the NMEs. However, these NMEs are still developed in individual tumor types, e.g., lung cancer, colorectal cancer (CRC), and gastric cancer, based on the anatomic origination of these tumors. Furthermore, anti-cancer drugs are nearly always tested in adult patients before they are developed in pediatric patient population. The recent development of anti-programmed cell death-1 (PD-1) antibodies, pembrolizumab (Keytruda) and nivolumab (Opdivo), and a pan-tropomyosin receptor kinase (TRK) inhibitor, larotrectinib (LOXO-101), has established a new paradigm in anti-cancer drug development. By applying biomarkers and focusing clinical development in well-defined and rare patient population, these drugs demonstrated unprecedented and unequivocal clinical efficacy to gain regulatory approval agnostic of tumor types, and simultaneously in both adult and pediatric patient populations.

## Anti-PD-1 antibodies (pembrolizumab and nivolumab) in MSI-H and dMMR solid tumors

The development of pembrolizumab for the treatment of tumors with microsatellite instability-high (MSI-H) and deficient DNA mismatch repair (dMMR) took a non-traditional path. When anti-PD-1 antibodies were first tested in 19 patients with CRC, only 1 responded [1, 2]. This single patient who responded to anti-PD-1 treatment had tumors with the MSI-H genotype and experienced a complete response (CR) lasting more than 3 years at the time of report [3]. It was hypothesized that dMMR resulted in increased somatic mutations found in this patient’s tumors which could subsequently be recognized by the patient’s own immune system. Immune checkpoint blockade by anti-PD-1 antibodies can further enhance the immune response.

The hypothesis that MSI-H and dMMR tumors contain abnormalities that affect the proper repair of DNA inside the cell was further investigated in an investigator-initiated phase 2 study (KEYNOTE-016) [4]. The study enrolled 41 patients with progressive metastatic carcinoma with or without dMMR to investigate the clinical activity of pembrolizumab, an anti-PD-1 immune checkpoint inhibitor. The MSI Analysis System (Promega, Madison, WI, USA) was utilized to assess the mismatch repair (MMR) status in tumors through the evaluation of selected microsatellite sequences that are particularly prone to copying errors when MMR is compromised. The co-primary endpoints, the immune-related objective response rate (irRR) and the 20-week immune-related progression-free survival (irPFS) rate, were 40% (4 of 10 patients) and 78% (7 of 9 patients) for patients with dMMR CRCs, and 0% (0 of 18 patients) and 11% (2 of 18 patients) for patients with MMR-proficient CRCs. The median progression-free survival (mPFS) and overall survival (mOS) were not reached at the time of report in the cohort with dMMR CRCs but were 2.2 and 5.0 months in the cohort with MMR-proficient CRCs [hazard ratio for disease progression or death, 0.10 (*P* < 0.001); hazard ratio for death, 0.22 (*P* = 0.05)]. Furthermore, patients with dMMR non-colorectal tumors had responses similar to those of patients with dMMR CRCs [irRR, 71% (5 of 7 patients); 20-week irPFS rate, 67% (4 of 6 patients)]. Whole-exome sequencing revealed a drastic difference in the number of somatic mutations, 1782 per dMMR tumor, as compared to 73 per MMR-proficient tumor (*P* = 0.007), and high somatic mutation loads were associated with prolonged progression-free survival (PFS) (*P* = 0.02). These data from this small phase 2 trial support the hypothesis that dMMR tumors are more responsive to PD-1 blockade than MMR-proficient tumors and form the basis for subsequent clinical trials leading to the registration of pembrolizumab for the treatment of MSI-H and dMMR tumors.

In addition to KEYNOTE-016 (*n* = 58), two prospective studies (KEYNOTE-158, *n* = 19; KEYNOTE-164, *n* = 61) were conducted to further confirm the efficacy of pembrolizumab in MSH-I and dMMR tumors [5]. Additionally, patients with MSI-H or dMMR solid tumors enrolled in two other trials (KEYNOTE-12, *n* = 6; KEYNOTE-28, *n* = 5) were retrospectively identified, and their responses were included in the analysis.

As listed in the Keytruda package insert, a total of 149 patients with MSI-H or dMMR cancers were identified from a total of 415 patients across these five uncontrolled, open-labeled, multi-cohort, multi-center, single-arm trials [6]. According to the prescribing information, the approval was based on efficacy observed in patients whose tumors were determined as MSI-H or dMMR either prospectively or retrospectively, either by the local lab or by a central lab, either by polymerase chain reaction (PCR) or immunohistochemistry (IHC) tests. The majority of these patients (135/149) was prospectively determined for their MSI-H or dMMR tumor status using local laboratory-developed PCR tests for MSI-H or IHC tests for dMMR. The remaining 14 of the 149 patients were retrospectively identified with MSI-H by testing tumor samples using a central laboratory-developed PCR test. Of these 149 patients across 15 tumor types, 47 had dMMR, 60 had MSI-H, and 42 had both. An overall response rate (ORR) of 39.6% was observed, including 7.4% CR and 32.2% partial response (PR). The median duration of response had not been reached at the time of approval [95% confidence interval (CI), 1.6+ to 22.7+ months] with 78% of the patients having response duration ≥ 6 months.

On May 23, 2017, the US Food and Drug Administration (FDA) granted accelerated approval to Keytruda (pembrolizumab) for the treatment of adult and pediatric patients with unresectable or metastatic solid tumors that have been identified as having MSI-H or dMMR. This is a historical approval as the first time a cancer treatment is approved based on a common biomarker rather than the anatomic location in the body where the tumor originated.

It should be noted that the FDA approval is an accelerated approval based on these five uncontrolled, open-labeled, multi-cohort, multi-center, single-arm trials. A confirmatory phase 3 trial (KEYNOTE-177) is ongoing and will form the basis to support a full approval in the future. In this phase 3 trial, 270 patients with MSI-H or dMMR stage IV CRCs who have not received any prior treatment for their cancer, e.g., front-line patient population, will be randomly assigned to 200 mg of pembrolizumab every 3 weeks or 1 of 6 chemotherapy regimens of the investigator’s choice. Treatment is to continue until disease progression, unmanageable toxicity, or the completion of 35 cycles (pembrolizumab only).

Similarly, nivolumab, also a PD-1 inhibitor, has shown efficacy on MSI-H/dMMR tumors too. The phase 2 Checkmate-142 study evaluated nivolumab at 3 mg/kg in patients with MSI-H or dMMR metastatic CRC [7]. Overall, 32% patients (24 of 74) responded to nivolumab, including 2.7% (*n* = 2) with a complete response. Among patients who had progressed after receiving prior treatment with fluoropyrimidine-, oxaliplatin-, or irinotecan-based chemotherapy (*n* = 53), 28% responded to nivolumab. On August 1, 2017, the FDA granted an accelerated approval for nivolumab as a treatment for patients with MSI-H or dMMR metastatic CRC after progression on standard chemotherapy.

## Larotrectinib (LOXO-101) in NTRK-fusion solid tumors

Oncogenic TRK fusions directly induce cancer cell proliferation and activate various downstream signaling pathways. These TRK fusions occur rarely, but in a diverse spectrum of histological tumor types. Larotrectinib (LOXO-101) is the first selective small-molecule pan-TRK inhibitor. Larotrectinib blocks the ATP-binding site of the TRK family of receptors with 2–20 nmol/L cellular potency against TRKA, TRKB, and TRKC. The first evidence of larotrectinib was shown in the phase 1 dose escalation study NCT02122913 [8]. Using an in situ proximity ligation assay, a lamin A/C (*LMNA*)-neurotrophic tropomyosin receptor kinase 1 (*NTRK1*) gene fusion encoding a functional LMNA-TRKA fusion oncoprotein was detected in the tumor of a 41-year-old woman with soft tissue sarcoma metastatic to the lung. Upon receiving larotrectinib treatment, this patient underwent rapid and substantial tumor regression, achieving PR after only 1 cycle of treatment and near CR of the largest tumor lesion after 4 cycles of treatment, with an accompanying improvement in pulmonary dyspnea, increase in oxygen saturation, and decrease in plasma tumor marker carcinoembryonic antigen-125 (CA-125).

At the 2017 American Society of Clinical Oncology (ASCO) meeting, the first set of clinical data consisting of an integrated dataset from three studies was reported [9]. Patients with NTRK-fusion solid tumors were enrolled to the adult (NCT02122913, *n* = 8) and pediatric (NCT02637687, *n* = 12) phase 1 trials, and a phase 2 trial that enrolled adult/adolescent patients (NCT02576431, *n* = 35). TRK-fusion status was prospectively determined by a variety of local testing methods, including DNA sequencing, RNA sequencing, next-generation sequencing (NGS), fluorescent in situ hybridization (FISH), and IHC analyses. The majority of patients were dosed with larotrectinib at 100 mg twice a day on a continuous 28-day schedule. Investigator-assessed ORR according to the Response Evaluation Criteria in Solid Tumors (RECIST) version 1.1 was the primary objective. Secondary endpoints included duration of response and safety. A total of 55 patients (12 pediatrics and 43 adults; 4 months to 76 years old), all with TRK-fusion tumors, were enrolled (median prior treatments = 2). Fusions involved NTRK1 (*n* = 25), NTRK2 (*n* = 1), and NTRK3 (*n* = 29), and other 14 unique fusion partners.

Seventeen unique tumor types were treated: salivary cancer (*n* = 12), sarcoma (*n* = 10), infantile fibrosarcoma (*n* = 7), lung cancer (*n* = 5), thyroid cancer (*n* = 5), colon cancer (*n* = 4), melanoma (*n* = 4), cholangio cancer (*n* = 2), gastrointestinal stromal tumors (*n* = 2), and others (*n* = 4). For the 50 patients with confirmatory response data available, the ORR was 76% (95% CI 62%–87%; 12% CR and 64% PR) with responses observed in 12 tumor types, and occurred regardless of age, the presence of *NRTK* gene, or fusion partner. At the time of data report, 93% of responding patients or 75% of all patients remained on treatment or underwent surgery with curative intention. A median duration of response had not been reached, with 79% of responders remaining on treatment without progression at 12 months after treatment. The most common treatment-related adverse events (AEs) were fatigue (38%), dizziness (27%), and nausea (26%). Treatment-related AEs were most of grade 1 or 2, with few of grade 3. Only 7 (13%) patients required dose reductions, and all maintained tumor responses on reduced dose levels. No patient discontinued larotrectinib due to treatment-related AEs. NTRK solvent front mutations (TRKA G595R and TRKC G623R) were detected in 5 out of 6 patients who developed acquired resistance to larotrectinib treatment. Two of these patients were successfully treated with LOXO-195, a second-generation TRK inhibitor to address acquired resistance.

From these three single-arm, uncontrolled trials, larotrectinib has demonstrated unequivocal and durable antitumor activity in TRK-fusion cancers, across a wide range of tumor types, in both pediatric and adult patients, and was well-tolerated. Larotrectinib could be the first targeted therapy developed in a tissue type-agnostic manner to address patients with NTRK-fusion tumors. Similar to pembrolizumab, the dataset consisting of three trials was intended to support regulatory approval.

## Common features of clinical development of tumor type-agnostic therapy

A few common features shared by these three programs, pembrolizumab and nivolumab for patients with MSI-H and dMMR tumor, larotrectinib for patients with TKR-fusion tumors, include the speed of clinical development; tumor type-agnostic biomarker-guided patient selection; as well as parallel development in adult and pediatric patients.

The development of pembrolizumab for patient populations with MSI-H and dMMR tumors got its first hint in a phase 1 study when a CRC patient derived a CR from nivolumab treatment [3]. The group in Johns Hopkins University followed up with this clinical observation quickly with a phase 2 investigator-initiated study to further investigate the role of PD-1 blockade in treating patients with MSI-H and dMMR CRC or other tumors [4]. When the data from this phase 2 study were reported at the ASCO meeting in 2015, a company-sponsored phase 3 trial was already underway [10].

In the meantime, regulatory agency also showed unprecedented flexibility in working with the sponsors to expedite the clinical development, review, and ultimate approval of tumor type-agnostic indication labels. For example, when the first dataset was generated, the US FDA granted breakthrough designation encouraging the sponsor to further investigate pembrolizumab in these relatively rare patient populations.

During the review period, the US FDA granted pembrolizumab Priority Review designation for the indication, a regulatory framework that enables FDA to take action on an application within 6 months where the agency determines whether the drug, if approved, would significantly improve the safety or effectiveness of treating, diagnosing, or preventing a serious medical condition. Furthermore, the US FDA accommodated the submission of an integrated dataset pooled from five uncontrolled, single-arm clinical trials. In some trials, patients were prospectively identified to have MSI-H or dMMR cancers, whereas in other trials, a subgroup of patients were retrospectively identified as having MSI-H or dMMR cancers. Equally unprecedented, a total of 15 unique cancer types were treated among 149 patients enrolled across these 5 clinical trials, ranging from colorectal, endometrial to other gastrointestinal cancers. Lastly, the review of pembrolizumab for this new indication in patients with MSI-H and dMMR tumors was based on ORR and duration of response, both are only potential surrogate markers for measuring the ultimate clinical benefit—overall survival. The clinical efficacy observed in the 149 patients who were treated with pembrolizumab in the trials were unequivocal, 40% of patients had CR or PR. For 78% of those patients, the response lasted 6 months or longer. At the time of approval, further studies were underway to verify anticipated clinical benefits of pembrolizumab in additional patients with MSI-H or dMMR tumors. The development of nivolumab in MSI-H and dMMR metastatic CRC also took a similar path as that of pembrolizumab.

It is also worth noting that this new tumor type-agnostic indication in patients with MSI-H and dMMR tumors was approved after pembrolizumab had already obtained regulatory approvals for multiple indications including melanoma, non-small cell lung cancer (NSCLC), head and neck squamous cell cancer (HNSCC), classical Hodgkin lymphoma (cHL), and urothelial carcinoma. Similarly, nivolumab has already been previously approved for treating NSCLC, melanoma, renal cell carcinoma, HNSCC, cHL, and urothelial carcinoma. The safety profiles of both pembrolizumab and nivolumab have been well established to support a favorable benefit:risk ratio of these medicines in treating patients with MSI-H and dMMR tumors.

A different development path was taken for larotrectinib. The molecule was designed to specifically target tumors with TRK-fusion and started its development specifically in patients with tumors dependent on signals from NTRK-fusion events. Loxo Oncology (South San Francisco, CA, USA) designed the development path for larotrectinib by prospectively select patients with these TRK-fusion tumors, in both pediatric and adult populations, in a tissue-agnostic fashion, and proactively seeking regulatory interactions to gain orphan indication and breakthrough designations to facilitate clinical development and regulatory approval. Another TRK-focused program, Ignyta Inc.’s entrectinib, is taking the same tissue-agnostic development approach.

## Precision medicine in practice

### Measurement methods and classification of MSI

MSI testing classifies tumors into MSI-H when 30% or more of the repeats are unstable, MSI-low (MSI-L) if fewer than 30% of the repeats are unstable, or microsatellite stable (MSS) if no repeats are unstable [11]. MSI testing is conducted using either DNA- or IHC-based methods by targeting the sequencing of MMR genes or their protein products. MSI testing detects an abnormal number of microsatellite repeats, indicating that cancer likely arose from cells with defective MMR genes. PCR-based MSI testing amplifies a patient’s tumor DNA at several microsatellite sites and compare with normal DNA [12]. MSI testing can profile either the Bethesda markers (often 2-mononucleotide and 3-dinucleotide microsatellite loci) or 5-mononucleotide markers as recommended by the National Cancer Institute/International Collaborative Group on Hereditary Non-polyposis Colorectal Cancer (NCI/ICG-HNPCC). This method has 80%-91% sensitivity among tumors with mutL homolog 1 (*MLH1*) or mutS homolog 2 (*MSH2*) mutations and 55%–77% sensitivity among tumors with mutS homolog 6 (*MSH6*) or PMS1 homolog 2 (*PMS2*) mutations [11]. Based on the composition of the MSI panel, specificity may reach 90%. However, MSI testing is time-consuming and expensive. More simplified methods are being developed using a single-mononucleotide marker, CAT25, and reported high specificity and sensitivity for identifying tumors with dMMR; however, these new methods would need to be evaluated in various ethnic populations to ensure consistent results [13, 14].

On the other hand, IHC assays detect the presence or absence of protein products encoded by MMR genes, of which a missing protein suggests a mutation in the gene that codes for that protein. Most MSI-H tumors loss protein expression for at least 1 MMR gene. IHC determination of such protein expression is helpful for identifying the corresponding genes and in turn target MSI testing for mutation analysis [15]. IHC testing has a sensitivity of 83%, regardless of the MMR gene involved, with a specificity of 89% [11]. It is convenient, inexpensive, and may detect loss of MSH6 protein that can be missed via MSI testing, therefore is a complementary testing strategy for MSI testing [16].

Therefore, MSI testing and IHC are often used synergistically to detect cases that may be missed by either test alone; however, they can be used separately under individual clinical barriers such as cost and specimen availability [17].

### MSI testing in clinic

MSI-H tumors are most commonly found in colorectal, endometrial, and gastrointestinal cancers, but are also sporadically seen in other cancers.

MSI occurs in approximately 15% of sporadic CRCs and 22%–33% of endometrial cancer, and has been reported in 5% or less of bladder, prostate, breast, renal cell, pancreatic, biliary duct, gastric, gastroesophageal junction, small intestinal, small cell lung, thyroid cancers, sarcoma, and retroperitoneal adenocarcinoma [12, 13]. This distribution of MSI-H events among different tumor types was confirmed in two recent studies [18, 19]. The first study grouped tumor types into categories based on the frequency of MSI, from colorectal (20%) and endometrial (22%–33%) to cervical (8%) and esophageal (7%) to skin and breast cancers (0%–2%) [18]. Another recent study evaluated MSI status and the number of unstable loci in 18 tumor types. MSI tumors were identified in 14 of these cancer types, with the highest proportion of MSI-H cases observed in endometrial, gastric, rectal, and colon cancers [18].

### The future of MSI and dMMR as a biomarker

The approval of pembrolizumab as the first anticancer therapies based on biomarker status rather than tumor anatomic origin has highlighted the need to assess MSI and dMMR status in a broad patient population. These tumor type-agnostic drug approvals, along with other drugs being developed in a tumor type-agnostic fashion, demonstrate the value of conducting multipanel NGS. However, the cost and reimbursement barriers may still remain in rendering the adaptation of a broad use of NGS in daily oncology practice, especially in community practice.

Although MSI testing has become an established part of the molecular examinations for patients with CRC where approximately 15% of sporadic CRCs are MSI-H, it has yet to become part of the routine testing for other tumor types due to the low rate of MSI-H events in these tumors. However, MSI status may still play a crucial role in determining the best treatment choices. As demonstrated in pembrolizumab-treated patients with MSI-H and dMMR status, immunotherapy such as anti-PD-1 or anti-programmed death-ligand 1 (PD-L1) checkpoint antibodies may represent a viable and effective treatment option for these patients with endometrial, ovarian, skin, gastric, urinary tract, upper gastrointestinal tumors, glioblastomas, and lymphomas [18]. Such effective treatment evidence will encourage the broader adaptation of MSI and MMR assays in these rare patient populations.

For these tissue-agnostic approaches, awareness and testing are essential to success. Recent FDA approvals of NGS panels, such as Memorial Sloan Kettering Center Center’s MSK-IMPACT, Foundation Medicine Inc.’s *FoundationOne,* and Thermo Fisher Scientific Inc.’s *Oncomine*, should help. MSK-IMPACT, which stands for integrated mutation profiling of actionable cancer targets, is a high-throughput NGS platform. In addition to detect actionable mutations in various cancer-driving kinases, one component of the FDA’s authorized intended use for MSK-IMPACT is MSI detection. These multiplex NGS platforms make it feasible to detect key tumor defects using limited tumor specimens.

## Caution—tumor type-agnostic drug development is not a universal model

Although pembrolizumab, larotrectinib, and entrectinib have demonstrated the value of tumor type-agnostic approach to anti-cancer drug development, such histology-independent development model is not going to universally work for all new anti-cancer drugs. In fact, most new anti-cancer drugs will likely still need to follow the traditional tumor type-specific development path.

Roche’s VE-BASKET trial of *Zelboraf* (vemurafenib), a phase 2 trial, enrolled patients with any type of non-melanoma cancer who had v-Raf murine sarcoma viral oncogene homolog B1 (*BRAF*) V600 mutations. ORR was 42% (95% CI 20%–67%) and mPFS was 7.3 months (95% CI 3.5–10.8 months) in the cohort of NSCLC patients. Similar ORR of 43% (95% CI 18%–71%) and mPFS of 5.9 months (range 0.6–18.6 months) were seen in the cohort with Erdheim–Chester disease or Langerhans’-cell histiocytosis. In contrast, only anecdotal responses were observed among patients with pleomorphic xanthoastrocytoma, anaplastic thyroid cancer, cholangiocarcinoma, salivary duct cancer, ovarian cancer, and clear-cell sarcoma and among patients with CRC who received vemurafenib and cetuximab. These results demonstrate that histologic context still plays a key role in determining clinical efficacy. Conventional treatment based on organ site, with molecular subtypes, cannot be entirely replaced by molecular nosology, e.g., *BRAF*-mutated cancers [20].

In the phase 2 SUMMIT basket trial, 124 patients with v-erbB2 avian eryhtorblastic leukemia viral oncogene homolog 2 (*HER2*) mutations and 17 patients with *HER3* mutations across 21 unique tumor types were treated with neratinib, a HER2/3 inhibitor. In the *HER2*-mutant cohort, clinical responses were observed in patients with breast, cervical, biliary, salivary, and non-small-cell lung cancers, but not in those with bladder cancer and CRC [21]. On the other hand, no activity was observed in the *HER3*-mutant cohort. These results again suggest that specific activation mutations do determine the responses to targeted therapy. However, such determination is still dependent, at least in some instances, on histology context.

Tumor type-agnostic development may therefore only be applicable to drugs with high clinical response rate and in rare tumor types.

## Conclusions

With the approval of pembrolizumab for patient with MSI-H and dMMR tumors, tumor type-agnostic therapy has already become a reality. The efficient development of TRK inhibitors, larotrectinib and entrectinib, demonstrates the utility of such tumor type-agnostic development path in developing medicines for rare tumor types. The recent approval of multiplex NGS platforms will further facilitate the adaptation of these medicines in clinical practice. However, tumor type-agnostic approach should still be taken, both during drug development and in clinical practice, with great caution so that tumor histology context is not lost.
